# RNA Pol III promoters—key players in precisely targeted plant genome editing

**DOI:** 10.3389/fgene.2022.989199

**Published:** 2023-01-04

**Authors:** Sakshi Dharmendra Kor, Naimisha Chowdhury, Ajay Kumar Keot, Kalenahalli Yogendra, Channakeshavaiah Chikkaputtaiah, Palakolanu Sudhakar Reddy

**Affiliations:** ^1^ International Crops Research Institute for the Semi-Arid Tropics, Hyderabad, Telangana, India; ^2^ Biological Sciences and Technology Division, CSIR-North East Institute of Science and Technology (CSIR-NEIST), Jorhat, Assam, India; ^3^ Academy of Scientific and Innovative Research (AcSIR), Ghaziabad, India

**Keywords:** CRISPR/Cas9, RNA pol III promoters, U6 and U3 snRNA promoters, TATA-box, USE, synthetic promoter

## Abstract

The clustered regularly interspaced short palindrome repeat (CRISPR)/CRISPR-associated protein Cas) system is a powerful and highly precise gene-editing tool in basic and applied research for crop improvement programs. CRISPR/Cas tool is being extensively used in plants to improve crop yield, quality, and nutritional value and make them tolerant to environmental stresses. CRISPR/Cas system consists of a Cas protein with DNA endonuclease activity and one CRISPR RNA transcript that is processed to form one or several short guide RNAs that direct Cas9 to the target DNA sequence. The expression levels of Cas proteins and gRNAs significantly influence the editing efficiency of CRISPR/Cas-mediated genome editing. This review focuses on insights into RNA Pol III promoters and their types that govern the expression levels of sgRNA in the CRISPR/Cas system. We discussed Pol III promoters structural and functional characteristics and their comparison with Pol II promoters. Further, the use of synthetic promoters to increase the targeting efficiency and overcome the structural, functional, and expressional limitations of RNA Pol III promoters has been discussed. Our review reports various studies that illustrate the use of endogenous U6/U3 promoters for improving editing efficiency in plants and the applicative approach of species-specific RNA pol III promoters for genome editing in model crops like Arabidopsis and tobacco, cereals, legumes, oilseed, and horticultural crops. We further highlight the significance of optimizing these species-specific promoters’ systematic identification and validation for crop improvement and biotic and abiotic stress tolerance through CRISPR/Cas mediated genome editing.

## Introduction

Promoters are the key regulatory elements present upstream of the transcription start site that controls the transcription of a gene through the involvement of the TFs and RNA polymerases. Promoters play a critical role in regulating gene expression that can be greatly modified by identifying and applying specific promoter systems, such as constitutive or inducible, for genetic manipulation of an organism for a desired trait/s. Promoters are classified as constitutive, tissue-specific, stage/temporal-specific, or inducible based on their ability to control gene expression (Kummari et al., 2020). However, recent advancements in transgene expression studies have led to the development of synthetic promoters consisting of repeats of *cis*-elements to drive the desired gene of interest. A synthetic promoter should be optimized for precise specificity, immediate inducibility, versatile applications, and efficient editing (Ali and Kim, 2019). Promoters are also classified as pol II and pol III, based on their ability to recognize RNA polymerases. The pol II promoter is the region that involves the binding of RNA polymerase II to initiate DNA transcription (Venter and Botha, 2010).

On the other hand, polymerase III aids the exclusive transcription of small non-coding RNAs, including 5S rRNA, tRNAs, and another type 3 RNAs such as the U6 snRNA (Cramer et al., 2008). The promoter elements are present internally in type 1 and type 2 genes of the polymerase III promoters. In contrast, the type 3 Pol III promoters typically utilize the upstream regulatory elements with a distinct +1 transcription start site and distinguished stretches of “thymine” as a termination signal (Schramm and Hernandez, 2002). Several studies have been conducted to understand the polymerase activity of the commonly used type 3 Pol III promoters, such as U6, 7SK, and H1. Recent studies of Gao et al. (2018), provide functional evidences of Pol II and Pol III competing for usage of promoter like human H1 promoter (Myslinski et al., 2001; Gao et al., 2018).

Nevertheless, type 3 Pol III promoters have found their application in the expression of small RNAs, like short hairpin RNAs in RNAi, and guide RNAs in the breakthrough CRISPR/Cas system. Typically, the Pol III type 3 promoters, like U6, comprises a ∼21 bp proximal sequence element, and a ∼8 bp TATA box located upstream of the transcription start site (+1) are reported to be conserved among species (Dahlberg and Lund, 1988). However, RNA polymerase specificity may be attributed to the minor sequence differences in the 3’ end of the proximal sequence element (Hernandez et al., 2007).

U6 promoters have reportedly been used to drive small hairpin RNA (shRNA) expression in vector-based RNA interference (Nie et al., 2010), and in identifying and characterizing U6 promoters from the genome of *Plutella xylostella* for enabling genome editing in non-model organisms (Huang et al., 2017). However, both the U3 and U6 promoters have been highly exploited in plants for efficient guide RNA activity (Belhaj et al., 2013). The U3 and U6 promoters in plants have a discrete transcription start site with adenine (A) and guanine (G), respectively. Therefore, the consensus sequence of A(N)19-22 for the U3 promoter and G(N)19–22 for the U6 promoter is considered ideal for designing the guide sequences of the sgRNAs (Belhaj et al., 2013). The U6 snRNAs contribute to the intron splicing of pre-mRNA in the nucleus (Li et al., 2007), while the U3 snRNAs are involved in pre-rRNA processing (Marz and Stadler, 2009).

**FIGURE 1:**
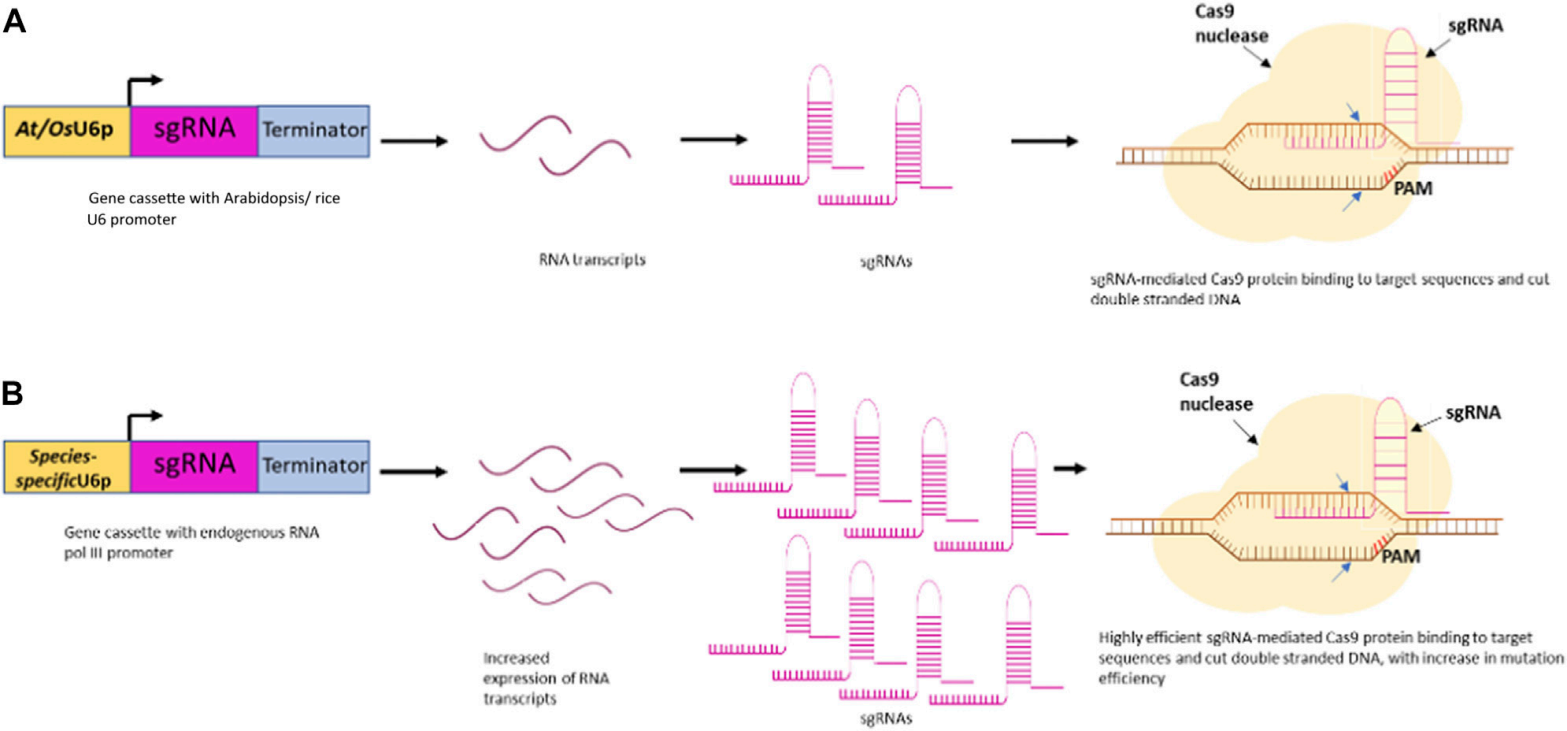
Schematic illustration of RNA Polymerase III promoter facilitating high-efficiency CRISPR/Cas9-mediated plant genome editing. **(A)** sgRNA expression under *At/OsU6* promoter in plant **(B)** sgRNA expression under species-specific promoter in plant leads to increased expression and higher gene editing efficiency.

The revolutionary platform of genome editing with CRISPR/Cas has unlocked opportunities to explore the genetic makeup of all plant species. The sole influence of the Cas protein and the single guide RNA can profoundly affect the editing efficiency of the CRISPR/Cas9 system. The plant promoters like U3 and U6 have established their place for driving the expression of single guide RNA due to their proficiency in producing high levels of sgRNA, with a length of ∼200 nucleotides (Shockey, 2020). The commonly used promoters in plant genome editing are the *Arabidopsis* (AtU3 and AtU6) and rice (OsU3 and OsU6) promoters used specifically for dicots and monocots, respectively (Lowder et al., 2015; Ma et al., 2015). Moreover, studies on the applicability of species-specific Pol III promoters provided some significant insight into the improved editing efficiencies due to increased sgRNA expression (Sun et al., 2015; Ng and Dean, 2017; Long et al., 2018) (Figure 1).

These type 3 Pol III promoters have a dual polymerase activity, making their usage more attractive for the concurrent expression of a small RNA and a protein. This expression system can complement the CRISPR/Cas9 genome-editing system, which involves the Cas9 protein and the single guide RNA. Ren et al. (2022) proposed that using Pol III promoter would abate the complexities involving transgene cassettes and aid the construction of viral vectors with limited packaging capacity. Nevertheless, exploring the pol III promoters from different plant species and their characterization can lead to milestones in the field of CRISPR/Cas9 genome editing (Ren et al., 2022).

## Structural features of Pol III promoters

In all eukaryotes, the genes are transcribed by one of the three RNA polymerases: RNA Pol I, II, and III. Each type of RNA polymerase performs a different function of transcribing. The RNA Pol I is responsible for transcribing a single set of ribosomal RNA genes with a single recognizable promoter structure. The second is RNA Pol II, which transcribes the protein-coding (mRNA) genes along with some small nuclear RNA (snRNA) genes, while the third class of polymerase, RNA Pol III, transcribes small, non-coding RNA set of genes like 5S rRNA and tRNA. RNA pol III transcribed genes which are involved in cellular metabolic processes like t-RNA processing, mRNA splicing, and protein synthesis (Schramm and Hernandez, 2002). The RNA Pol II and III promoters share common features, such as a similar TATA-box as a recognition site, while have some distinct features like the presence of poly-T (thymine) tail at 3′end as terminator whereas, Pol II promoters have *cis*-elements as their 3′-terminal signal and such Poly-T sites present endogenously in the sequence (Richard and Manley, 2009). There are three sub-classes of RNA Pol III promoter, namely type 1, 2, and 3, which are classified based on the position of the promoter with respect to the gene and the existence of the TATA-box. The type 1 and 2 promoters are gene-internal and TATA-less box, which assists in the transcription of 5S rRNA genes and Adenovirus (Ad2) VAI gene, tRNA set of genes, respectively (Fowlkes and Shenk, 1980; Bogenhagen et al., 1982; Schramm and Hernandez, 2002). The third class of Pol III promoter is distinct from the other two sub-classes by the presence of TATA-box and promoter sequence being present at the 5′ flanking ends or upstream to the transcription start site (+1TSS). This class of promoter includes U1 to U6, signal recognition particle (SRP), mitochondrial RNA processing (MRP) snRNAs, H1, etc. (Kunkel and Pederson, 1988; Baer et al., 1990; Topper and Clayton, 1990). The type 3 Pol III promoters have conserved regions as that of snRNA Pol II promoters, like Distal Sequence Element (DSE) and Proximal Sequence Element (PSE) in mammals or Upstream Sequence Element (USE) in plants, in addition to TATA box located at 30 bp upstream to the TSS (+1). The TATA-box has all required information to cluster together the elements for RNA Pol III transcription initiation (Mitchell et al., 1992; Roberts et al., 1995; Wang and Stumph, 1995; Schramm and Hernandez, 2002).

**FIGURE 2:**
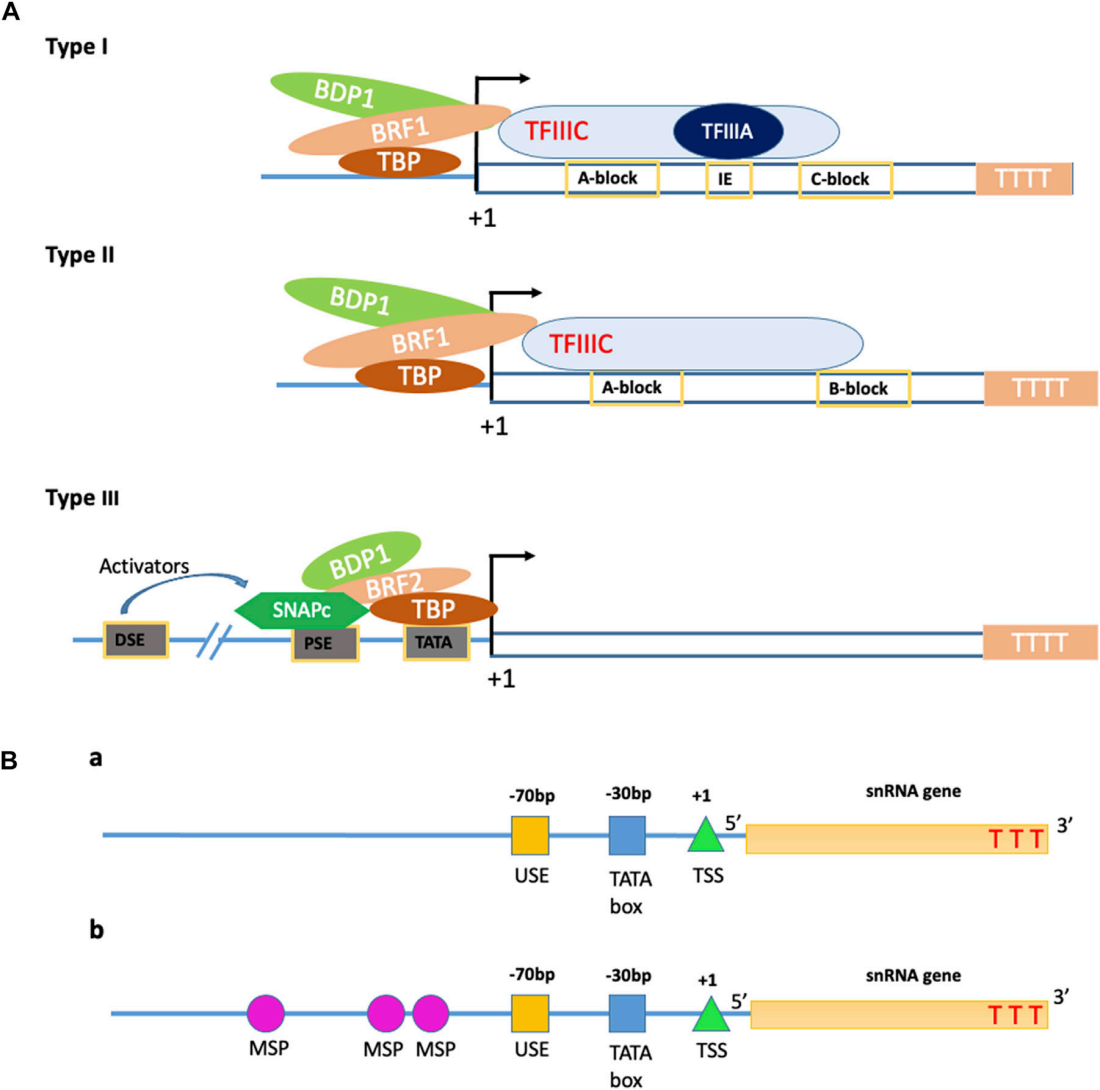
Structural properties of the Pol III promoters. **(A)** Schematic representation of the structure of different types of Pol III promoters. B-double prime 1 (BDP1), B-related factor 1 (BRF1), TATA-box binding protein (TBP), B-related factor 2 (BRF2), proximal sequence element (PSE), Distal sequence element (DSE), small nuclear RNA activating protein complex (SNAPc); +1—Transcription start Site; TTTT—terminator site. **(B)** The structural arrangements of plant Pol III promoters in a dicot (a) and monocot (b) plants. The arrangement of regulatory elements, namely, TATA box, Upstream sequence element (USE), and Transcription start site (TSS) of type 3 Pol III promoters in dicot plants (a). The same regulatory elements with the addition of monocot-specific promoters (MSPs) in monocot Pol III promoters (b). TTT—Thymine.

In plants, the two basal promoter elements required for Pol III transcribed snRNA genes are the −70 bp highly conserved plant snRNA gene-specific element, USE (consensus RTCCCACATCG) and −28 to −30 bp TATA-box (Figure 2A) (Marshallsay et al., 1990; Waibel and Filipowicz, 1990). The U6 and U3 snRNA gene promoters have the USE element placed one helical turn closer to the TATA box than that in Pol II specific genes, which have the USE and TATA box positioned four helical turns apart (Marshallsay et al., 1990). In dicots, the sequences present upstream to USE have no significance in snRNA gene transcription but, an extra element located upstream to the USE in monocots, known as the monocot-specific promoter element (MSP), increases the efficiency of transcription (Figure 2B). These MSP(s) (consensus, RGCCCR) is present in one to three copies in the monocot snRNA gene promoter region. In monocots, the efficiency of snRNA gene transcription is determined by the strength of the MSP element/s present in the promoter region while that of dicot is measured using the quality of the USE element (Marshallsay et al., 1992). The AT-rich region of RNA Pol III resembles the TATA box found in Pol II, but the AT-rich box distinguishes Pol III promoters from that of mRNA promoters (Pol II) by initiating the transcription in downstream of the “forward” TATA box, whereas transcription initiated by Pol III is in downstream of the “reverse” TATA sequence (Mattaj et al., 1988; Lobo and Hernandez, 1989; Waibel and Filipowicz, 1990; Wang and Stumph, 1995). The similarities and differences between RNA Pol II and Pol III promoters are given in Table 1.

**TABLE 1:** Similarities and differences between RNA Pol II and RNA Pol III promoters.

RNA polymerase II	RNA polymerase III
It transcribes mRNA encoding genes, long and some small non-coding RNAs genes	It transcribes short non-coding RNA genes, like 5S rRNA, tRNAetc.
Pol II promoters have such Poly-T sites present internally	Pol III promoters have Poly-T sites at the 3′end
AT-rich region that resembles the TATA box in Pol II promoters	AT-rich box acts as TATA box to distinguish from Pol II promoter
RNA pol II is sensitive to 1 μg/ml of α-amanitin	RNA pol III is sensitive to 10 μg/ml of α-amanitin

## Functional characteristics of the RNA Pol III promoters

The snRNA promoters have different characteristics which differentiate them from other classes of gene coding promoters. The U-snRNA class (U1, U2, U4, and U5) are transcribed by RNA Polymerase II, while genes like H1, U3, and U6 snRNAs are transcribed by RNA Polymerase III (Dahlberg and Lund, 1988). The genes encoding snRNAs in plants and vertebrates are unique in a way that some transcribed by Pol II and some by Pol III, but both classes of genes have similar promoter elements (Murphy et al., 1987; Filipowicz et al., 1990). These set of genes have their promoters for the recruitment of RNA polymerase III. In monocots and dicots, the Pol III promoters are used for transcription of *U3* and *U6* snRNA genes. These Pol III promoters are generally used in expression of small nuclear RNA, short hairpin RNA, and guide RNA in the CRISPR/Cas9 genome editing system (Ma et al., 2014). In most of the CRISPR/Cas9 constructs, the RNA polymerase III -type 3- U3 or U6 promoters are used for expression of sgRNA in monocots and dicots. Further, Pol III promoters are extensively used for expression of polycistronic tRNA-sgRNA construct involved in multiple gene-targeted genome editing (Jiang et al., 2013). These Pol III promoters need a very specific 5′ nucleotide, U6 promoter requires 5′-Guanine (G) and U3 needs 5′-Adenine (A) to start the transcription (Jiang et al., 2013). Thus, specificity can be increased by addition of specific nucleotide at 5′end of the target sequence or the gRNA sequences. These U6 and U3 promoters drive the expression of gRNAs in plants but may not always work for all targeted genes due to the absence of spatial and temporal specific control, as it is ubiquitously expressed in all tissues and at all stages of growth and development (Gao and Zhao, 2014; Xie et al., 2015).

CRISPR/Cas9 plant genome editing system uses two sets of RNA polymerases. Expression of Cas9 gene under RNA polymerase II promoter while the sgRNA cassette is driven by the RNA Polymerase III (U6 or U3 promoters) (Jiang et al., 2013). The two types of promoters control the co-expression of Cas9 and gRNA. While targeting the expression of multiple gRNAs in a single cassette, there will be a corresponding number of Pol III promoter sequences, further leading to increased cassette size, which is the limitation for cloning into the vectors (Li et al., 2013; Nekrasov et al., 2013; Shan et al., 2013). For efficient cloning of multiple guide RNAs, Collonnier et al. (2017) suggested using a different combination of promoters such as a U3 promoter for one sgRNA and a U6 promoter for the second sgRNA in the same vector backbone to avoid the hairpin structure formation and smooth DNA synthesis (Collonnier et al., 2017).

## Activation of RNA polymerase III promoters

The transcription initiation leads to the polymerase complex formation in the promoter region. Protein factors or TFs that recognize the sequence motifs of RNA polymerase III transcribed genes are well studied in yeast and animals. The multi-subunit complex for activation of RNA polymerase III promoter includes TFIIIC, A and B boxes, TFIIIB, and TATA-binding protein (TBP) in yeast and vertebrates (Orioli et al., 2012). The recruitment of RNA Pol III to the promoter region in a plant is sketchily known. The Pre-initiation complex (PIC) assembly is formed about 50 bp upstream region of the transcription start site (TSS) of the pol III-transcribed gene. Thus, it is the prime surface of interaction with TFIIIB. The TFIIIB is responsible for the recruitment of the RNA polymerase III enzyme. The TBP is another protein involved in Pol III-dependent transcription and is a component of TFIIIB. The TATA box element of the U6 snRNA genes is a core promoter element for the transcription of RNA Pol III (Zhang et al., 2011). This TATA box governs the TSS selection by Pol III, with the aid of the A-box-bound τA sub-complex of TFIIIC. This τA is said to be extended within which TBP chooses the TATA-like sequence. This TATA-like sequences provide surface for assembly of TFIIIB and thus recruits TFIIIB and initiates the process of transcription downstream of 5′end of TATA element (Orioli et al., 2012).

The tCAAca sequence is another core promoter element in fungi, and plant Pol III transcribed genes are involved in TSS selection by RNA Polymerase III. In this tCAAca sequence, the uppercase letters indicate the least variable positions, and the first A is the TSS (Giuliodori et al., 2003; Yukawa et al., 2011; Zhang et al., 2011). The shared mechanistic characteristic between the tCAAca sequence and the initiator by RNA Polymerase II is the presence of two pyrimidines before A of TSS, this being the common feature between Pol II and Pol III transcription elements (Orioli et al., 2012).

## Assembly of transcriptional initiation complex of Pol III promoters

RNA polymerase III is responsible for the bulk of transcriptional activity, including all the important non-coding RNAs (whole set of transfer RNAs, U6 spliceosomal RNA, and 5S ribosomal RNA) (Abascal-Palacios et al., 2018). The RNA polymerases III enzyme share numerous subunits with RNA polymerase II, but it identifies a different set of promoters with distinct transcription factor proteins (Shen, 2019). The most notable and uncommon aspect of pol III promoters is that most of them require sequence components downstream of the transcription start site (+1), i.e., they have promoters entirely within the genes (Shen, 2019). The typical and classical RNA polymerase III promoters are type 1 (e.g., 5S rRNA gene), type 2 (e.g., tRNA gene), and type 3 (promoter of the *Homo sapiens* U6 snRNA) (Kummari et al., 2020).

Type 1 promoters of RNA polymerase III require two internal sequence elements for efficient transcription, an A block located from +50 to +70 and a C block from +80 to +90, and an intermediate element (IE) between blocks A and B (Figure 2A). Type 2 of RNA polymerase III promoters comprises two sequence blocks (A and B) present within the gene transcription region and are very conserved (Goodfellow and White 2005). Distinct from type 1 and 2, the type 3 promoter of RNA polymerase III (example: U6 snRNA gene) falls under the non-classical category. They have a transcription factor binding site upstream of the transcription start site (+1 site), and it consists of a TATA box (located amid −30 to −25 from +1 Site) and another upstream control sequence element named proximal sequence element (PSE), and finally upstream to PSE is a distal sequence element (DSE) (Goodfellow and White 2005; Arimbasseri and Maraia, 2016).

Transcription with RNA polymerase III takes 3 general steps: initiation, elongation, and termination. Positioning of eukaryotic RNA polymerase III enzyme to the transcription start site (TSS) requires many transcription factors that work synergistically. To initiate transcription on type 1 promoters, the RNA polymerases III complex relies on a different set of transcription factors (TFIIIA, TFIIIB, and TFIIIC) as it has less affinity for promoter sequence elements (Goodfellow and White 2005; Park et al., 2017). Briefly, TFIIIC interacts with internal promoter sequences (block A and B) and recruits the TFIIIB complex. TFIIIA binds specifically to the intermediate element (IE) of type 1 and recruits TFIIIC to its site and RNA polymerase to promote transcription initiation from the +1 site. In this case, the TFIIIB is a complex of three proteins, TBP (TATA-box binding protein), BDP1 (B double prime 1), and BRF1 (B-related factor 1) (Park et al., 2017). The assembly of transcription factors on type 2 promoter (e.g., tRNA) differ from that of type 1 promoters. The TFIIIC of the type 2 promoters (same set of protein as type 1) recognizes and binds to the A and B blocks of type 2 internal promoter and recruits the TFIIIB (B double prime 1, BDP1; B-related factor 1, BRF1; and TATA-box binding protein, TBP) and RNA polymerase to the transcription start site (Figure 2A) (Arimbasseri and Maraia, 2016; Park et al., 2017).

In the case of type 3 promoters (e.g., U6 snRNA gene), assembly of a transcription factor on the promoter sequence occurs upstream of the transcription start site (TSS). Here SNAPc (small nuclear RNA activating protein complex), an activating protein complex, binds to the upstream promoter element, proximal sequence element (PSE) to promote the TFIIIB recruitment and RNA polymerase III loading for transcription initiation. In this case, TFIIIB consist of TBP (TATA-box binding protein), BDP1 (B double prime 1), and BRF2 (B-related factor 2) (Arimbasseri and Maraia, 2016; Park et al., 2017).

RNA polymerase III has a high and steady small nuclear RNA transcriptional activity, accounting for approximately 40% of total RNA, which is validated by the fact that Pol III promoter has a primary role in RNA-guided genome editing strategies like CRISPR/Cas technology (Paule and White, 2000). The guide RNA used in the CRISPR/Cas9 technology is usually driven by RNA polymerase III (Ma et al., 2014). Another striking fact is that RNA polymerase III has defined sites for transcription initiation and termination, making them good candidates for genome editing techniques like CRISPR/Cas (Brummelkamp et al., 2002). Promoters like U6 and U3 are reported to work efficiently in plants where RNA Pol III transcribes them ubiquitously and constitutively to express guide RNAs (Li et al., 2013; Bortesi and Fischer, 2015). Types and features of RNA Pol III promoters are given in Table 2.

**TABLE 2:** Types of RNA Pol III promoters.

	Type 1	Type 2	Type 3
Transcribing genes	5S rRNA	VAI gene	U3/U6 snRNA
Location of promoter with respect to gene	Gene-internal	Gene-internal	Gene-external
TATA-box	Absent	Absent	Present
Upstream Sequence Element (USE)	Absent	Absent	Present
Conserved domains	A, E and B boxes	A and B box	TATA-box and USE
Transcription factors	T.F IIIA, T.F IIIC	T.F IIIC	T.F IIIB, T.F IIIC

## Synthetic RNA Pol III promoters

A synthetic promoter is a sequence of DNA which is artificially designed in order to regulate the expression of the target gene. The *cis*-regulatory element sequences of a promoter that exist in nature are used as fundamental blocks for synthesizing these artificial promoters. These can be created using rational design or ligation (Roberts, 2011). Synthetic promoters are important in studying the *cis*-motif elements’ orientation, strength-weight, and position to understand gene regulation *in vivo.* These strategies can be used in designing of the expression cassette for target genes in genome editing technology (Venter and Botha, 2010). Hao et al. (2020) modified the active rice U3 and U6 promoters by shortening the 5′ sequences but retaining the USE and TATA box elements and the native MSPs, along with adding two to three artificial MSPs in the upstream region of USE to increase the transcriptional efficiency. Synthetic promoters were used to improve the efficiency of gene transcription for activating the *GUS* reporter gene in pco-dCas9-VP64 coupled with multiple sgRNAs (Lowder et al., 2015). These synthetic promoters were designed to check the functionality of the pco-dCas9-VP64 transcriptional activator and pco-dCas9-3X repressor (Lowder et al., 2015). The same strategy was used to develop the Orthogonal Control System (OCS) based on orthogonal synthetic promoters driven by the Artificial Transcription Factor (ATF). The constitutively expressing Pol III promoters can be synthetically controlled to express in a specific tissue, thus widening the use of OCS for targeted genome editing. The synthetic promoter needs its own transcription factor to be constructed and characterized (Kar et al., 2022). They preferentially drive the expression of Cas9, in Arabidopsis egg cell (Durr et al., 2018). Also, in yeast *Yarrowia lipolytica*, the single gene disruption efficiency of 92% and more was obtained due to synthetic hybrid promoters, RPR1′-tRNA^Gly^, SCR1′- tRNA^Gly^ and SNR52′- tRNA^Gly^ under native RNA Pol III promoter (Schwartz et al., 2016). Löbs et al. (2017), used CRISPR/Cas9 system from *S. pyogenes* for *Kluyveromyces marxianus* genome editing using hybrid RNA Pol III promoters like RPR_1_-tRNA^Gly^, SCR1′- tRNA^Gly^ and SNR52′- tRNA^Gly^ hybrid promoters for knocking out alcohol dehydrogenase (ADH) and alcohol-O-acetyltransferase genes.

## Applications of U6/U3 promoters in CRISPR/Cas-mediated genome editing

The plant species-specific Pol III promoters like U6 and U3 have been extensively used for increased sgRNA levels to achieve efficient editing using the CRISPR/Cas technology. In the last two decades, there have been several reports addressing the use of species-specific U3/U6 promoters in targeting certain traits in cereals, legumes, oilseeds, and horticultural crops, the details of which are discussed hereunder. Endogenously identified species-specific RNA Pol III promoters to enhance the genome editing efficiency are represented in Table 3.

**TABLE 3:** Endogenously identified species-specific RNA Pol III promoters to enhance genome editing efficiency.

Type	Plant	Common name	Promoter (U3/U6)	Target gene	References
*Monocots*	*Oryza sativa*	Rice	OsU3	*ADH2*	Mikami et al. (2015)
	*Zea mays*	Maize	ZmU3	Argonaute 18 and anthocyaninless genes	Char et al. (2017)
	*Sorghum bicolor*	Sorghum	SbU6	PDS, GDH7, kafirin, Apetela2	Massel et al. (2022)
	*Triticum aestivum*	Wheat	TaU6.1, TaU6.3	GFP	Zhang et al. (2019a)
	*Musa acuminata*	Banana	MaU6	PDS, Luciferase reporter	Zhang et al. (2022)
	*Phyllostachys edulis*	Mosa bamboo	PeU3	PDS	Huang et al. (2022)
*Dicots*	*Arabidopsis thaliana*	Arabidopsis	AtU6-1	*BON*	Li et al. (2014)
			AtU6-26	*St16DOX* and SI*IAA9*	Nakayasu et al., 2018; Ueta et al., 2017
			AtU6-29	EOD3	Khan et al. (2020)
			AtU3	*EOD3*	Khan et al. (2020)
	*Nicotiana benthamiana*	Tobacco	NbU6	*PDS*	Li et al. (2014)
	*Camelina sativa*	False flex or linseed dodder	CsU3	*FAD*	Morineau et al. (2017)
	*Malus domestica*	Apple	MdU3	*PDS*	Charrier et al. (2019)
	*Fragaria vesca*	Wild strawberry	FveU6-2	Auxin biosynthesis gene (TAA1), auxin response factor 8 (ARF8)	Zhou et al. (2018)
	*Vitis vinifera*	Grapevine	VvU3	PDS	Ren et al. (2022)
	*M. domestica*	Apple	MdU6	*PDS* and *TFL1*	Charrier et al. (2019)
	*C. sativa*	False flex or linseed dodder	CsU6	*FAD*	Morineau et al. (2017)
	*Cichorium intybus*	Chicory	CiU6-1	*PDS*	Bernard et al. (2019)
	*Coffea canephora*	Coffee tree	CcU6	*PDS*	Breitler et al. (2018)
	*Vigna unguiculata*	Cowpea	VuU6	*SPO11*, *Rec8* and *OSD1*	Juranic et al. (2020)
	*Glycine max*	Soyabean	GmU6	Glyma06g14180, Glyma08g02290, Glyma12g37050	Sun et al. (2015)
	*Gossypium hirsutum*	Cotton	GhU6 3.3	*PDS*	Long et al. (2018)
	*Hevea brasiliensis*	Rubber tree	HbU6	*PDS*	Dai et al. (2021)
	*Lotus japonicus*	Lotus	LjU6-1	*LjSYMRK*	Wang et al. (2016)
	*Medicago truncatula*	Alfalfa	MtU6	*PDS*	Meng et al. (2017)
	*Picea glauca*	White spruce	PaU6	*DXS1*	Cui et al. (2021)
*Bryophyte*	*Marchantia polymorpha*	Liverwort	MpU6-*1pro*	Auxin response factor (*AF1*)	Sugano et al. (2014)
*Gymnosperm*	*Cryptomeria japonica*	Japanese cedar	CjU6	*CjChll*	Nanasato et al. (2021)

In a study conducted by Li et al. (2013a), protoplast transient expression system was used for exploring the use of sgRNA:Cas9 technology. The plant codon-optimized SpCas9 and the sgRNAs was transcribed from the hybrid constitutive 35SPPDK promoter and the Arabidopsis U6 Polymerase III promoter, respectively. The sgRNAs were designed for targeting the *A. thaliana* gene*s* viz., phytoene desaturase (*PDS3*), flagellin sensitive (*AtFLS*2), and the *Nicotiana benthamiana* ortholog of *AtPDS3* (*NbPDS*). Moreover, the authors targeted two members of the Arabidopsis RACK1 (Receptor for Activated C Kinase 1) family with multiple sgRNAs expressed under the U6 promoter, thereby ensuring targeted mutagenesis and gene knockout (Li et al., 2013a). In a contemporary study by Nekrasov et al. (2013), they used the sgRNA:Cas9 system for targeting the *PDS* gene in *N. benthamiana* with the sgRNA expressed under an Arabidopsis U6 promoter (Nekrasov et al., 2013). Similarly, the targeted mutation in *PDS* and *PDR*-type transporter genes was achieved through the CRISPR/Cas platform where the chimeric guide RNA was driven by the AtU6-26 promoter (Gao et al., 2015). Other research in Arabidopsis includes the targeted mutagenesis of endogenous DNA glycosylase genes *ROS1* and *DME* using sgRNA driven by the AtU6 promoter (Miki et al., 2018). Successful heritable homozygous mutations were also reported in the T_2_ generation by using the Arabidopsis U6-26 promoter (Fauser et al., 2014; Feng et al., 2014).

Jiang et al. (2013) demonstrated the CRISPR/Cas9 mediated genome editing in immature embryos of sorghum where the sgRNA was expressed under the rice U6 promoter (Jiang et al., 2013). Shan et al. (2013) reported the design of two sgRNA, SP1 and SP2 for disrupting the rice phytoene desaturase gene *OsPDS* along with specific sgRNAs for targeting the *OsBADH2*, *Os02g23823*, and *OsMPK2* genes in rice using the rice endogenous U3 promoters (Shan et al., 2013). Moreover, the wheat U6 promoter was used to drive the sgRNA for targeting the wheat ortholog of barley MLO protein, *TaMLO.* In an aim to target the Maize *IPK* gene, involved in the phytic acid biosynthetic pathway, Liang et al. (2014) designed the sgRNA to express under the Maize U3 promoter. They confirmed the mutation of Inositol 1,3,4,5,6-pentakisphosphate 2-kinase gene in *Zea mays* using the CRISPR/Cas genome editing (Liang et al., 2014). Svitashev et al. (2016) reported DNA-free genome editing in maize by targeting four genes viz., male fertility genes (*MS26* and *MS45*), liguleless1 (*LIG*) and acetolactate synthase (*ALS2*). Under the expression of maize U6 promoter, the *in vitro* transcribed gRNAs and the purified Cas9 protein were pre-assembled to initiate the targeted mutagenesis (Svitashev et al., 2016). Very recently, Char et al. (2020), through CRISPR/Cas9 system demonstrated targeted mutagenesis in two endogenous genes of Sorghum, *SbFT* and *SbGA2ox5,* responsible for flowering time and plant height. The designed sgRNAs were driven by two different rice U6 promoters, and the induced mutations were passed on to the T_1_ generation (Char et al., 2020). In another contemporary study by Liu et al. (2020), the efficiency to drive single-guide RNA in wheat was observed for three different promoters from rice (OsU6a) as well as wheat (TaU3 and TaU6), through *Agrobacterium*-mediated transformation. TaU3 promoter was found to be a better choice than OsU6a or TaU6 for driving the expression of sgRNA in wheat. A high editing efficiency of 80.5% was achieved by the optimized SpCas9 system using TaU3 and two sgRNAs for targeted mutagenesis of two endogenous genes, *TaWaxy* (granule-bound starch synthase I) and *TaMTL* (MATRILINEAL, a pollen-specific phospholipase) (Liu et al., 2020). In the above usage of OsU6 or TaU6 promoter for sorghum, genome editing can be replaced by recently identified endogenous sorghum SbU6 promoters by Massel et al. (2022). They identified eight putative SbU6 promoters in the BTx623 genome and selected SbU6_2.3 and SbU6_3.1 promoters to target *β-kafirin* (major grain storage protein). Using SbU6_2.3 resulted in 80.0% of the mutation rate in the *β-kafirin* gene. Thus, endogenous pol III promoter employment leads to a higher and more efficient CRISPR/Cas editing system. (Massel et al., 2022).

In an attempt to demonstrate the application potential of CRISPR/Cas9 in a forage crop like *Medicago truncatula*, Michno et al. (2015) successfully mutated a GUS transgene in somatic cells of *M. truncatula* through root hair transformation, where the target guide RNA was expressed under the Arabidopsis U6 promoter (Michno et al., 2015). In subsequent research, Meng et al. (2017) targeted the second exon of the *phytoene desaturase* (*MtPDS*) gene using a sgRNA under the effect of the native MtU6 promoter (Meng et al., 2017). The *symbiosis receptor-like kinase* (*SYMRK*) gene is crucial for nodule and arbuscular mycorrhizal symbiosis in legumes. Targeted disruption of three targets of exon 2 of the *VuSYMRK* in Cowpea (*Vigna unguiculata*) through the CRISPR/Cas9 system was carried out by Ji et al. (2019). The respective gRNAs were designed to be expressed under the U6 promoter, resulting in approximately 67% mutagenesis (Ji et al., 2019). The *SYMRK* gene was also targeted for mutagenesis in *Lotus japonicus* along with three homologous leghemoglobin loci (LjLb1, LjLb2, LjLb3), the designed guide RNAs of which were placed under the effect of LjU6-1 promoter (Wang et al., 2016). In yet another study, Chen et al. (2020) established an ‘allele-aware chromosome-level genome assembly’ genome editing protocol in *Medicago sativa* L. The expression of the sgRNAs targeting the *PDS* and *PALM1* (encoding a Cys(2)His(2) zinc finger transcription factor) genes was driven by the MtU6 Polymerase III promoter (Chen et al., 2020).

Genome modification in soybean was demonstrated by Jacobs et al. (2015) by targeting the transgene Green Fluorescent Protein, a putative glucosyl-transferase endogenous gene (Glyma07g14530), and the orthologs of the *A. thaliana DDM1* gene (Glyma01g38150 and Glyma11g07220) (Jacobs et al., 2015). The single guide RNAs were driven by the *M. truncatula* U6.6 Polymerase III promoter. Michno et al. (2015) performed the hairy root transformation in soybean, where they designed the guide RNA to target the *Glutamine synthase (GS1)* and *chalcone-flavanone isomerase* (*CHI20*) genes under the effect of the Arabidopsis U6 promoter (Michno et al., 2015). To address the problem of seed shattering from mature fruits in tetraploid oilseed rape (*Brassica napus*), Braatz et al. (2017) used CRISPR/Cas9 construct by targeting two homologs of the *ALCATRAZ (ALC)* gene. The sgRNA was placed under the control of the Arabidopsis U6-26 promoter, where a single target sequence generated four *alc* mutant alleles in an edited T_1_ plant (Braatz et al., 2017). Contemporary studies with CRISPR/Cas9 gene editing also altered the fatty acid composition in *Camelina sativa* seeds by targeting *the FAD2* gene responsible for synthesizing polyunsaturated fatty acids. *C. sativa* *,* being an allohexaploid, the three homoeologous *FAD2* genes were targeted using the same sgRNA, which was driven by the Arabidopsis U6 promoter (Jiang et al., 2017). The same *FAD2* gene was modulated using the CRISPR/Cas9 system in *B. napus* cv. Westar and in peanut (*Arachis hypogaea* L.) using sgRNAs, driven by the Arabidopsis U6 promoter and *M. truncatula* U6 promoter, respectively (Okuzaki et al., 2018; Yuan et al., 2019). The enzyme lysophosphatidic acid acyltransferase (*LPAT*) aids the catalysis of fatty acid chains into 3-phosphoglycerate, thereby enhancing oil production. The *BnLPAT2* and *BnLPAT5* genes from *B. napus* were targeted using the Arabidopsis U6-26 promoter to drive the sgRNA expression further establishing their role in oil biosynthesis (Zhang K. et al., 2019). In another study, Di et al. (2019) analysed the effect of multiple *G. max* U6 promoters by targeting three genes, *Glyma03g36470*, *Glyma14g04180*, and *Glyma06g136900* through *Agrobacterium rhizogenes* infection, while Zhang Z. et al. (2019) tested the ECp-Cas9/gRNA system by targeting the *GmAGO7a* (Glyma.01G053100) and *GmAGO7b* (Glyma.02G111600) using the Arabidopsis U3 or U6 promoters to drive expression of each gRNA (Zhang Z. et al., 2019; Di et al., 2019).

Wang et al. (2015) used potato U6 RNA (StU6P) for initiating the expression of sgRNA *via Agrobacterium tumefaciens* mediated transient expression of *phytoene desaturase (PDS)* gene in *N. benthamiana.* They further transformed the CRISPR/Cas9 construct in potato to make stable mutations in the *StIAA2* gene encoding an Aux/IAA protein in potato (Wang et al., 2015). A contemporary study of genome editing was reported in cucumber (*Cucumis sativus* L.) by Chandrasekaran et al. (2016) (Chandrasekaran et al., 2016). Targeted disruption of the *eIF4E* gene (eukaryotic translation initiation factor 4E), was demonstrated through Cas9/sgRNA editing. Two sgRNAs, expressed under the effect of Arabidopsis U6 promoter, were designed to target two sites of the *eIF4E* gene. A successful CRISPR/Cas9 editing of the *flavanone-3-hydroxylase (F3H)* gene was performed in the carrot. Two single-guide RNA (gRNAs) was expressed in the CRISPR/Cas9 vectors under the effect of the Arabidopsis U3 promoter for obstructing the biosynthesis of anthocyanin (Klimek-Chodacka et al., 2018).

To study the effect of CRISPR/Cas9 genome editing in tomato, Brooks et al. (2014) constructed sgRNA for targeting the tomato homolog of Arabidopsis ARGONAUTE7 (*SlAGO7*) through *Agrobacterium*-mediated transformation. The sgRNAs, expressed under the effect of the Arabidopsis U6 promoter, were in duplicates in order to create large and well-defined deletions. The mutant plants had needle-like or wiry leaves as compared to the compound leaves in wild-type tomatoes (Brooks et al., 2014). First study for genome editing in apple was reported by Nishitani et al. (2016). Precise modification in the apple *phytoene desaturase* (*PDS*) gene was confirmed by the use of four sgRNAs that functioned under the effect of Arabidopsis U6 promoter, which resulted in an approximately 13.6% editing efficiency (Nishitani et al., 2016). In a proof-of-concept study by Charrier et al. (2019), the *PDS* and *Terminal Flower 1 (TFL1)* genes were successfully knocked-out in apple. Two guide RNAs were expressed with U3 and U6 apple promoters for targeted editing. Successful editing in the *MdPDS* gene was confirmed by distinctive albino phenotype in about 85% of the edited lines, while early flowering was observed in 93% of the edited lines where the *MdTFL1* was targeted (Charrier et al., 2019). Successful editing of the *auxin biosynthesis* (*TAA1*) and *auxin response factor 8 (ARF8)* genes of wild strawberry *Fragaria vesca* was achieved by Zhou et al. (2018). Two promoters viz., wild strawberry U6 promoter (FveU6-2) and Arabidopsis U6 promoter (AtU6-26) drove the expression of the sgRNAs targeting the two genes and both were reported to create efficient genome editing (Zhou et al., 2018). On the other hand, Kaur et al. (2018) successfully demonstrated the application of the CRISPR/Cas9 system in banana cv. Rasthali. Single guide RNAs targeting two phytoene desaturase genes (*RAS-PDS1* and *RAS-PDS2*), expressed under the rice U3 promoter, created albino phenotype and abnormalities in growth of the edited plants (Kaur et al., 2018). But Zhang et al. (2022) used endogenous MaU6 promoter instead of OsU6 promoter and also used banana codon-optimized Cas9, which increased the mutation frequency four times. Thus, developing a foundation for DNA-free genome editing technology in banana plants (Zhang et al., 2022). In a recent study, the efficacy and efficiency of four *Vitis vinifera* U3 and U6 promoters and two UBQ promoters in precise targeting of grape phytoene desaturase (PDS) gene was established by Ren et al. (2022). Further, the AtU6 promoter was replaced by the VvU6 promoter, for targeting multiple sgRNA and developing a multiplex genome editing system in grapes. The concurrent editing of the two genes viz., *TMT1* and *TMT2* (*tonoplastic monosaccharide transporter*) demonstrated the successful editing in grapes (Ren et al., 2022).

CRISPR/Cas9 technology has also been applied in ornamental flowers like Petunia, which also serve as a model system for comparative research. Zhang et al. (2016) targeted the *PDS* gene, with the sgRNA driven by the Arabidopsis U6 promoter, to make precise deletion in homozygous chromosomal fragment of the target gene. The lignocellulose biosynthesis process involves five genes viz., *C*_*3*_ *H*, *C*_*4*_ *H*, *4CL*, *CCR*, and *IRX* encoding coumarate 3-hydroxylase, cinnamate 4-hydroxylase, 4-coumarate: coenzyme a ligase, cinnamoyl coenzyme a reductase, and irregular xylem5 respectively (Zhang et al., 2016). Kui et al. (2017) designed 3 pairs of sgRNA for each gene, which were driven by the OsU3 promoter, to successfully apply the CRISPR/Cas9 tool for genome editing in *Dendrobium officinale* (Kui et al., 2017). The hexaploid, *Chrysanthemum morifolium* is an important ornamental plant where Kishi-Kaboshi et al. (2017) attempted genome editing using the CRISPR/Cas9 system. They targeted four sites of the transgene *CpYGFP* (yellowish-green fluorescent protein gene from *Chiridius poppei*) with four sgRNA under the control of the Arabidopsis U6 promoter (Kishi-Kaboshi et al., 2017).

Nanasato et al., 2021, performed targeted mutagenesis in Japanese cedar and used endogenous *CjU6* promoter to knock out the reporter *GFP* gene. They also mutated the endogenous magnesium chelates subunit I (*CjChlI*) gene using the CjU6 promoter to obtain the albino phenotype (Nanasato et al., 2021). Also, Dai et al. (2021), in the same year, developed an efficient method to validate the functionality of sgRNAs in rubber tree using endogenous five HbU6 promoters and reported the first plasmid-mediated genome editing report in *Hevea brasiliensis via* CRISPR/Cas9 system. This study targeted the *PDS* and *flowering time* (*FT*) related genes. The first report of an immature embryo plant regeneration system and genetic transformation system in *Phyllostachys edulis*, a monopodial bamboo species using two PeU3 promoters and targeting the *PePDS1* and *PePDS2* genes. The usage of endogenous pol III promoters led to higher editing efficiency (35%–39%) than editing with the OsU3 promoter (Huang et al., 2022). White spruce is one of the major sources of timber and pulpwood, having high economic and ecological importance. Cui et al. (2021) successfully knocked out the *DXS1* gene using *the PaU6* promoter in CRISPR/Cas9 toolbox to produce a high frequency of chimerism (Cui et al., 2021).

## Conclusion

This review on RNA Polymerase III promoters in plants has illustrated the importance of the type 3 RNA Pol III promoters in specifically creating mutations in targeted gene editing using CRISPR/Cas system. These U3/U6 promoters require mainly two elements for its activity viz., TATA-box and USE. Monocot-specific promoters require extra element upstream to USE known as MSPs to increase the transcriptional efficiency. Not much is known about the Pre-initiation complex (PIC) formation of snRNA Pol III promoters in plants thus, this area of research needs to be explored to understand more of the transcriptional factors and regulatory elements. The review highlights the U3 and U6 promoters and their application in various plant systems. Recent studies show that the use of endogenous RNA Pol III promoter that transcribes single or multiple guide RNAs in CRISPR/Cas9 system have increased the editing efficiency. Therefore, the researchers, presently are aiming to identify the species-specific U3/U6 promoters and to broaden the understanding of transcriptional assembly for more specific and efficient genome editing.
